# Seed Amplification Assays for Parkinson's Disease: A Review of α‐Synuclein Assays in Body Fluids and Tissues

**DOI:** 10.1111/jnc.70435

**Published:** 2026-05-16

**Authors:** Weijie Kong, Katsuya Satoh, Mika Inada Shimamura, Tetsuya Maeda, Kenta Takahashi, Masanori Kurihara, Atsushi Iwata

**Affiliations:** ^1^ Department of Health Sciences, Unit of Medical and Dental Sciences Nagasaki University Graduate School of Biomedical Sciences Nagasaki Japan; ^2^ Division of Neurology and Gerontology, Department of Internal Medicine, School of Medicine Iwate Medical University Iwate Japan; ^3^ Department of Neurology and Stroke Tokyo Metropolitan Geriatric Hospital and Institute of Gerontology Tokyo Japan

**Keywords:** α‐synuclein (α‐syn), Parkinson's disease (PD), RT‐QuIC, seed amplification assays (SAAs)

## Abstract

The pathological core of Parkinson's disease and related synucleinopathies involves the misfolding and aggregation of α‐synuclein. Seed amplification assays (SAAs) have revolutionized the detection of pathological α‐synuclein by enabling highly sensitive and specific identification of seeding activity. Cerebrospinal fluid (CSF)‐based real‐time quaking‐induced conversion (RT‐QuIC) demonstrates exceptional diagnostic accuracy for sporadic Parkinson's disease (PD) and dementia with Lewy bodies (DLB), with sensitivity reaching 93.3%–94.6% and pooled specificity of 94% (95% CI: 0.92–0.96), consistent with the meta‐analysis of 21 core CSF studies. Alternative samples such as skin and intestinal tissues offer diagnostic accuracy up to 94.1%, providing less invasive options. These assays can distinguish conformational differences between Parkinson's disease/Lewy body dementia and multiple system atrophy (MSA), revealing potential for differential diagnosis through strain typing. In prodromal screening, SAAs show remarkable utility, with positivity rates exceeding 80% in idiopathic rapid eye movement sleep behavior disorder (iRBD) cohorts, indicating detection years before clinical diagnosis. Despite these advances, current limitations include small‐sample sizes in many studies, insufficient multicenter validation, and lack of standardized protocols affecting interlaboratory consistency. Future efforts should focus on establishing standardized procedures, integrating digital biomarkers, and validating these technologies across diverse populations and disease stages to facilitate widespread clinical implementation. This study systematically evaluates the diagnostic performance of α‐synuclein SAAs across biological samples, their role in differential diagnosis, and their potential in prodromal prediction and disease monitoring.

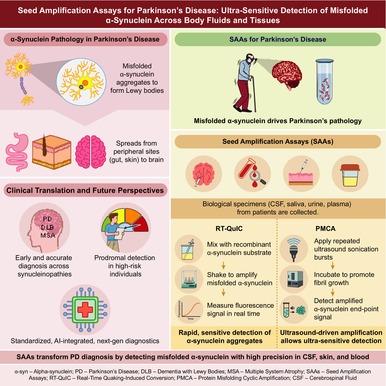

## Graphical abstract

**Background:** Parkinson's disease (PD) diagnosis is hindered by late motor symptom onset and low specificity of clinical assessments. This systematic review and meta‐analysis evaluates α‐synuclein seed amplification assays (SAAs) for PD and related synucleinopathies. **Main Results:** SAAs, including real‐time quaking‐induced conversion (RT‐QuIC) and protein misfolding cyclic amplification (PMCA), show high diagnostic accuracy via cerebrospinal fluid (sensitivity 93.3%–94.6%, specificity 97.5%–100%), minimally invasive skin biopsy (accuracy 94.1%), and noninvasive blood samples (sensitivity ~80.5%). SAAs also enable differential diagnosis and prodromal prediction. **Implications:** SAAs bridge the noninvasive gap in synucleinopathy diagnosis, facilitating early intervention and clinical translation.

**Abbreviations**
α‐syn: α‐synuclein
AD: Alzheimer's disease
CBS: Corticobasal syndrome
CNS: central nervous system
CVD: cerebrovascular disease
DAT‐PET: dopamine transporter positron emission tomography
DLB: dementia with Lewy bodies
ELISA: enzyme‐linked immunosorbent assay
ES‐QuIC: extracellular vesicle‐based QuIC
FFPE: formalin‐fixed paraffin‐embedded
HCs: healthy controls
IP‐QuIC: immunoprecipitation‐enhanced QuIC
IRB: Institutional Review Board
iRBD: idiopathic rapid eye movement sleep behavior disorder
LBD: Lewy body disease
MIBG: metaiodobenzylguanidine
MSA: multiple system atrophy
Nano‐QuIC: nanoparticle‐enhanced RT‐QuIC
NCs: neurological controls
NfL: neurofilament light chain
PD: Parkinson's disease
PDD: Parkinson's disease dementia
PMCA: protein misfolding cyclic amplification
PRISMA: Preferred Reporting Items for Systematic Reviews and Meta‐Analyses
PROSPERO: International Prospective Register of Systematic Reviews
PSP: progressive supranuclear palsy
qSAA: quantitative seed amplification assay
QSAA: quiescent seed amplification assay
QUADAS‐2: Quality Assessment of Diagnostic Accuracy Studies 2
RT‐QuIC: real‐time quaking‐induced conversion
SAAs: seed amplification assays
SOPs: standard operating procedures
UPDRS III: Unified Parkinson's Disease Rating Scale III

## 1 Introduction

Parkinson's disease (PD) (Hatano et al. 2024; Bellomo et al. 2022) is the second most common neurodegenerative disease following Alzheimer's disease. Its typical pathological features are the progressive loss of dopaminergic neurons in the midbrain substantia nigra and the formation of intracellular Lewy bodies (Carrazana et al. 2025; Iranzo et al. 2021). The core component of Lewy bodies is misfolded and aggregated α‐synuclein (α‐syn) (Sharma and Dhamija 2025) The aggregation of pathological α‐syn into Lewy bodies is the defining neuropathological feature of a spectrum of disorders collectively termed Lewy body disease (LBD) (Schalkamp et al. 2024).

This umbrella term primarily encompasses three clinical entities: PD, which is characterized by predominant motor symptoms; Parkinson's disease dementia (PDD), when dementia emerges in the context of well‐established PD; and dementia with Lewy bodies (DLB), where dementia precedes or coincides with the onset of parkinsonism. Although they differ in their initial clinical presentation and temporal sequence of symptoms, PD, PDD, and DLB share the same underlying pathological process (Malek et al. 2014).

For the purpose of this review, which focuses on the detection of pathological α‐syn, the term LBD will be used when discussing general principles applicable to this entire disease spectrum. The specific terms PD, PDD, or DLB will be used when referring to findings unique to those clinical presentations.

Notably, pathological α‐syn is present not only in the central nervous system (CNS) but also in peripheral tissues, such as skin, intestinal tract, and salivary glands (Beach et al. 2010), as well as circulating blood components including plasma (Gelpi et al. 2014). Multiple studies have validated the feasibility of detecting pathological α‐syn in skin biopsy samples across diverse ethnic populations (Böttner et al. 2012), supporting the “dual‐hit hypothesis” that the PD pathology may originate from the periphery and propagate to the CNS through the neural pathway (Gelpi et al. 2014; Böttner et al. 2012).

At present, PD is clinically diagnosed based on the presence of motor symptoms, such as tremor, rigidity, and bradykinesia. However, these manifestations often appear only after the occurrence of substantial neuronal damage, resulting in delayed diagnosis. In addition, PD needs to be distinguished from other synucleinopathies, such as multiple system atrophy (MSA) and dementia with Lewy bodies (DLB) (Bougea et al. 2025; Kleinz et al. 2025), which show significant overlap in early clinical symptoms. Distinguishing these diseases based on clinical manifestations alone is difficult.

Therefore, the development of biomarkers that enable objective, early, and accurate diagnosis of PD and identification of related diseases before death has become an urgent priority in current neurodegenerative disease research (Sharma and Dhamija 2025). This demand is particularly prominent for the differential diagnosis of atypical parkinsonian disorders, where fluid‐based biomarkers have shown promising discriminatory value in recent research (Bougea et al. 2025).

Beyond single molecular biomarkers, the integration of digital biomarkers with molecular detection has also emerged as a promising direction, with digital risk scores showing high sensitivity in identifying individuals with α‐synuclein aggregation or dopaminergic deficit (Schalkamp et al. 2022).

As the core of the pathological cascade, misfolded α‐syn is an ideal molecule to achieve this objective. However, in the early or prodromal stages of the disease, such as idiopathic REM sleep behavior disorder (iRBD), the levels of pathological α‐syn in body fluids or tissues are extremely low, exceeding the detection limits of traditional immunodetection methods. However, in recent years, the seed amplification assay (SAA) technology has provided a new means to address this problem. Seed amplification assay (SAA) is an umbrella term for techniques that simulate prion‐like amplification of pathological α‐synuclein seeds, including the two most widely used methods: real‐time quaking‐induced conversion (RT‐QuIC) and protein misfolding cyclic amplification (PMCA). These representative techniques simulate the prion‐like amplification mechanism.

This process enables the exponential amplification of trace amounts of α‐syn “seeds” in vitro, thereby achieving highly sensitive detection, with recent studies further demonstrating the capability of SAA‐based methods to detect pathological seeds of multiple neurodegenerative disease‐related proteins, including α‐synuclein, tau, and prion protein, even on solid surfaces (Orrú et al. 2024). Traditional biomarker detection relies on the quantitative determination of total α‐syn concentration (such as using enzyme‐linked immunosorbent assay [ELISA] technology), but these methods have two major limitations: (1) lack of specificity—unable to distinguish physiological α‐syn from pathogenic misfolded aggregates; (2) insufficient sensitivity—the concentration of pathogenic α‐syn in the early or prodromal stage (such as iRBD) is extremely low, far exceeding the detection limit (Malek et al. 2014; Hatano et al. 2024).

The α‐syn‐SAA technology, by mimicking the prion‐like amplification mechanism, can specifically amplify trace pathogenic α‐syn “seeds” and achieve a paradigm shift from “nonspecific concentration determination” to “specific detection of pathogenic seeds” (Bellomo et al. 2022; Gilboa et al. 2024; Al‐Lahham et al. 2025). This technological breakthrough provides a core tool for solving the dilemma of early diagnosis of PD and differentiating related disease subtypes, and has also become the core direction of current research on biomarkers of synaptic prionopathies.

Previous studies have demonstrated that cerebrospinal fluid (CSF)‐based α‐syn RT‐QuIC has an extremely high diagnostic sensitivity of approximately 98% and a specificity of nearly 100% for both Parkinson's disease (PD) and dementia with Lewy bodies (DLB). Furthermore, this technique can distinguish the difference in amplification kinetics between PD/DLB and MSA‐derived α‐syn, demonstrating the potential of “strain typing” and providing a molecular basis for differential diagnosis (Bellomo et al. 2025).

The application scope of SAAs has been extended from CSF to peripheral tissues (e.g., skin and olfactory mucosa) and body fluids (Cao et al. 2019; Marek et al. 2025; Manne et al. 2020b; Kuang, Mao, Huang, et al. 2024) (e.g., blood and saliva), which can be easily obtained using minimally invasive methods and have broad prospects for clinical transformation. In prodromal screening, SAA positivity in the iRBD population suggests a high risk of progression to typical synucleinopathy (Rossi 2021; Brown et al. 2025), and emerging evidence has also linked subclinical Lewy body pathology to subtle cognitive changes in clinically unimpaired individuals (Palmqvist et al. 2023), providing a critical time window for early intervention and risk stratification.

Furthermore, in SAAs using tissue homogenates, the results vary depending on whether SDS or a different surfactant type is used during sample preparation. Notably, utilizing this difference enables straightforward differentiation between Lewy body dementia (LBD) and MSA.

Despite the promising prospects, SAA still faces challenges, including insufficient standardization of detection procedures, lack of systematic evaluation of the diagnostic performance of varying biological samples, and differences in the result interpretation between laboratories.

Moreover, although previous network meta‐analyses have compared the diagnostic performance of α‐syn‐SAAs across different biological samples (Zheng et al. 2023), systematic studies with updated evidence and standardized evaluation frameworks are still lacking. Therefore, this study aimed to systematically evaluate the diagnostic value of α‐syn‐SAAs in PD and related synucleinopathies, focusing on the following objectives:

1. To compare the diagnostic accuracy of α‐syn‐SAAs in different biological samples (e.g., CSF, skin, blood).
2. To evaluate the role of SAAs in the differentiation of PD from other forms of atypical parkinsonism (e.g., MSA, progressive supranuclear palsy [PSP]).
3. To summarize the application potential of SAAs in prodromal prediction and disease progression monitoring.

By sorting out existing evidence and pointing out the current limitations, this study aims to provide directions for future research and promote the standardization and clinical application of α‐syn‐SAAs.

## 2 Method

### 2.1 Meta‐Analysis Search Strategy

Prior to the initiation of this study, we focused on the meta‐analysis of α‐syn‐SAA diagnostic efficacy and did not complete prospective registration in PROSPERO (International Prospective Register of Systematic Reviews).

Recognizing the importance of research transparency for systematic reviews, we have subsequently supplemented the registration. According to the plan we had previously formulated (Project ID: 1186650 on the Prospero platform), the complete protocol is accessible via the PROSPERO database. This post hoc registration ensures that the study design and analysis plan are fully traceable.

After the initial meta‐analysis search, two independent researchers (Weijie Kong [W.K.] and Shimamura Mika [S.M.]) systematically screened the retrieved records in two stages.

Title/abstract screening: Studies obviously irrelevant to α‐syn‐SAA diagnostic research were excluded; Full‐text screening: For studies that passed title/abstract screening, the full texts were retrieved, and the two researchers independently verified whether they met all inclusion criteria (e.g., availability of raw diagnostic data, clear study design).

Any discrepancies between the two researchers during screening were resolved through discussion; if consensus could not be reached, a third senior researcher (Satoh) was consulted for arbitration. The consistency of screening results between the two researchers was evaluated using Cohen's kappa coefficient (*κ* = 0.89), indicating good inter‐rater reliability.

To ensure the transparency and reproducibility of the meta‐analysis screening process, we constructed a flow diagram in strict accordance with the Preferred Reporting Items for Systematic Reviews and Meta‐Analyses (PRISMA) guidelines, detailing the number of records at each selection stage. A total of 2876 relevant records were initially retrieved from the PubMed database, with the search timeframe restricted from January 2011 to October 2025. After removing 623 duplicate records, 2253 unique records underwent title and abstract screening.

Of these, 1941 records were excluded, with the primary reasons including nondiagnostic study design, irrelevant target disease (e.g., Alzheimer's disease without concomitant synucleinopathy), review articles, and preclinical animal studies.

Subsequently, 312 full‐text articles were retrieved for full eligibility assessment. Among them, 200 articles were excluded based on the predefined exclusion criteria, with the main reasons as follows: unavailability of raw diagnostic performance data (e.g., unreported sensitivity and specificity), sample size < 20 cases, non‐peer‐reviewed conference abstracts, and studies not using SAA‐based detection methods (e.g., ELISA for total α‐synuclein measurement). Finally, 112 eligible studies were included in the subsequent qualitative synthesis and quantitative meta‐analysis.

### 2.2 Selection Strategy Informed by the Meta‐Analysis

#### 2.2.1 Study Selection Criteria

Studies were included if they met all of the following criteria:

1. published in English,
2. included patients with PD or other synucleinopathies (e.g., DLB or MSA, which has been reported to show distinct heterogeneity in α‐synuclein seeding activity; Martinez‐Valbuena, Visanji, Kim, et al. 2022) as well as healthy controls and/or controls with other neurological diseases,
3. included all clinically relevant biological sample types that have been applied in α‐syn seed amplification assay (SAA)‐based diagnosis (CSF, skin tissue, peripheral blood, and saliva), regardless of pre‐analytical processing methods (e.g., exosome enrichment for blood samples),
4. employed seed amplification techniques (e.g., protein misfolding cyclic amplification SAA subtypes [RT‐QuIC/PMCA]) as detection methods, and
5. reported diagnostic sensitivity and specificity or other equivalent diagnostic accuracy indicators directly or indirectly.

#### 2.2.2 Exclusion Criteria

Studies that met any of the following criteria were excluded:

1. duplicate published studies;
2. systematic studies, meta‐analyses, letters, research protocols, case reports, case series, and conference abstracts (but their references will be searched for potentially eligible original studies);
3. studies with unavailable full text and incomplete data, from which diagnostic performance information could not be extracted; and
4. studies solely based on animal models or in vitro cell lines. As the standard cut‐off value of α‐syn‐SAA has not been established, the threshold of positive detection has not been prespecified.

### 2.3 Data Extraction and Quality Assessment

The extraction content included sample type, detection method, sensitivity, specificity, sample size, etc.

### 2.4 Statistical Analysis

A random effects model was used to analyze the diagnostic performance of varying samples. Youden's J (“sensitivity plus specificity − 1”) was calculated to assess the overall discriminatory power of each test. Furthermore, to comprehensively compare the performance of different assays, a Cut‐off Analysis was constructed to visualize the trade‐off between sensitivity (true positive rate) and 1 − specificity (false‐positive rate) across included studies.

**FIGURE 1:**
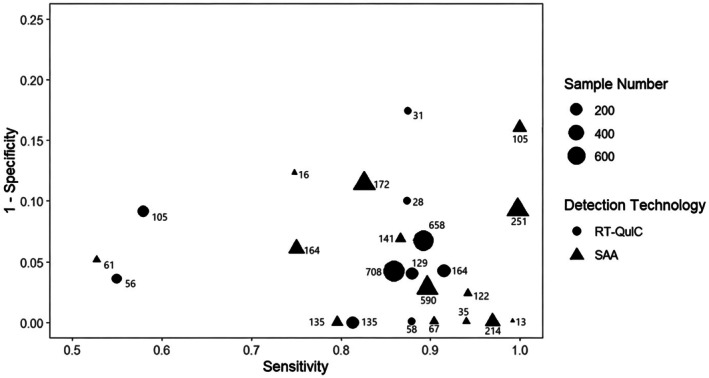
Diagnostic performance landscape of CSF α‐synuclein SAAs: a cut‐off analysis. Each data point represents an independent CSF‐based study mapped in the diagnostic space of sensitivity (*X*‐axis, 0.50–1.00) and 1 − specificity (*Y*‐axis, 0.00–0.25). Detection technologies are differentiated by shape: black circles (●) for RT‐QuIC assays and black triangles (▲) for other SAA subtypes. The size of each symbol is proportional to the study's sample size (categorized as small, medium, and large), with the exact number of cases annotated adjacent to the corresponding point. The pronounced clustering of studies—particularly large‐scale RT‐QuIC cohorts—in the high‐sensitivity (0.80–1.00) and low‐false‐positive region visually defines the optimal diagnostic threshold, underscoring the robust clinical utility of CSF α‐syn‐SAA for identifying Parkinson's disease and dementia with Lewy bodies.

Figure 1 quantifies the optimal cut‐off thresholds for CSF‐α‐synuclein seed amplification assays (SAAs) and real‐time quaking‐induced conversion (RT‐QuIC), with data points encoded by detection technology (black circles for RT‐QuIC, black triangles for SAA) and sized by study sample number (200/400/600 cases) to reflect statistical weight. For studies without direct access to raw data, a discrete data‐based cut‐off mapping method was employed to more truly reflect their diagnostic performance, focusing on the clustering of data points in the high‐sensitivity/low‐false‐positive region. All code were constructed using the R language environment RStudio and installation packages (meta, metafor, mada, lme4, shiny, ggplot2, forestplot, plotly, dplyr, readr, tidyr, igraph, netmeta) (R Core Team 2025; Schuler et al. 2025).

This code enables reproducibility of the statistical analyses, including data import, sample size calculation, and visualization of results (e.g., annotation of sample numbers adjacent to each data point in the Cut‐off plot).

### 2.5 Effect of Sample Type and Pretreatment on the Performance of α‐Syn‐SAA

Sample type comparison: Different biological samples have their own advantages and disadvantages in the detection of α‐syn seed amplification. CSF is currently the “gold standard” for diagnosis, with extremely high sensitivity, but its collection method is invasive and difficult to repeat (Bellomo et al. 2023).

Skin biopsy provides high specificity and can be verified through pathological methods, but this operation requires professional medical personnel to perform. The prominent advantage of blood/serum samples is that they are completely noninvasive and easy to obtain, and circulating α‐synuclein has long been explored as a potential plasma‐based biomarker for PD (Malec‐Litwinowicz et al. 2018; Zhao et al. 2022; Okuzumi et al. 2023), but their disadvantage is that the detection sensitivity is relatively low and is easily affected by the matrix. Saliva, as another noninvasive sample, is the easiest to collect, but it has problems of poor stability and insufficient related research (Vivacqua et al. 2016).

Furthermore, the preprocessing of the samples is a crucial step. Factors such as storage temperature, the number of freeze–thaw cycles, and the use of protease inhibitors can significantly affect the final test results, as demonstrated in previous technical validations of RT‐QuIC assays across multiple tissue and fluid types (Manne et al. 2019b).

### 2.6 In‐House Validation Study Patient Cohort

We retrospectively analyzed 287 patients with LBD and 600 controls recruited at Nagasaki University, Tokyo Medical Biobank and Iwate Medical University. The study was approved by the Institutional Review Board (IRB No. [R23‐031]). Written informed consent was obtained. Strict decontamination procedures were implemented throughout the experimental process to prevent cross‐contamination, given the high resistance of pathological α‐synuclein seeds to conventional inactivation methods (Pinder et al. 2021). SAA Protocol: CSF samples were analyzed using the RT‐QuIC protocol as previously reported (Kurihara et al. 2024).

## 3 Challenges in Diagnostic Accuracy

The clinical diagnosis of α‐synucleinopathies (α‐syn) has long relied on clinical phenotypic identification, and there is a lack of molecular means to directly detect pathological α‐syn, which makes it difficult to differentiate this type of disease from other neurodegenerative diseases, especially in the early stages of the disease, where insufficient diagnostic accuracy becomes an important bottleneck in clinical treatment (Sharma and Dhamija 2025).

### 3.1 Assessment Based on Meta‐Analysis

This study strictly followed the PRISMA guidelines for meta‐analysis and full‐process management, and the study protocol was registered on the PROSPERO International Prospective Registry Platform for systematic reviews (Project ID: 1186650) to ensure traceability of the study design and analysis protocol. A total of 2876 articles related to the diagnosis of α‐SYN‐SAA from January 2011 to October 2025 were initially retrieved from the PubMed database.

After removing 623 articles, the titles and abstracts of 2253 unique articles were screened. After excluding 1941 articles that were not related to the diagnosis of α‐syn‐SAA, full‐text evaluations were conducted on 312 articles. A total of 112 eligible studies were included for qualitative synthesis and quantitative meta‐analysis in accordance with the established criteria.

To ensure the methodological rigor and reproducibility of the meta‐analysis, this study clearly defined the predefined criteria for all excluded, among which Kurihara et al.'s (2024) study focused on the correlation analysis between α‐syn‐SAA sensitivity and metaiodobenzylguanidine (MIBG). The original diagnostic efficacy data such as sensitivity and specificity required for Parkinson's disease (PD) diagnosis were not provided, which did not meet the core inclusion criteria of “providing quantifiable diagnostic performance indicators” of this study.

Therefore, the decision was completely consistent with the PROSPERO registry protocol (Kurihara et al. 2024). The consistency of the meta‐analysis process was verified by Cohen's kappa coefficient as *κ* = 0.89, suggesting inter‐rater reliability between the two independent appraisers and reliable screening results. The comprehensive raw diagnostic data and methodological characteristics extracted from the 112 included studies, which form the quantitative foundation of this meta‐analysis, are detailed in Table 1 (Baiardi et al. 2025; Peña‐Bautista et al. 2023).

**TABLE 1:** Raw data from included studies for the diagnostic accuracy meta‐analysis of α‐synuclein seed amplification assays.

Author	Year	Tissues	Technology	Sensitivity	Specificity	Sens_SE	Spec_SE	TP	FP	FN	TN	Reference
Brown E G	2025	CSF	α‐Synuclein SAA	70%	—	—	—	182	78	—	—	Brown et al. (2025)
Christian D. Orrú	2025	CSF	α‐Synuclein SAA	91.40%	97.50%	1.5%	1%	321	6	30	233	Orrú et al. (2025)
Christian D. Orrú	2025	CSF	α‐Synuclein SAA	90.70%	94.10%	1.4%	1.70%	380	14	39	225	Orrú et al. (2025)
Baiardi, S	2025	CSF	α‐Synuclein SAA	81.60%	100%	5.5%	—	40	0	9	86	Baiardi et al. (2025)
Baiardi, S	2025	CSF	α‐Synuclein SAA	79.60%	100%	5.8%	—	39	0	10	86	Baiardi et al. (2025)
Kleinz	2025	CSF	α‐Synuclein SAA	100%	100%	—	—	2	0	0	1	Kleinz et al. (2025)
Rissardo J	2025	CSF	α‐Synuclein SAA	100%	83.90%	0%	3.80%	12	15	0	78	Rissardo et al. (2025)
Goldstein D S	2025	CSF	α‐Synuclein SAA	88%	83%	—	—	7	4	1	19	Goldstein et al. (2025)
Srivastava A	2022	CSF	PMCA	88.5%	96.9%	—	—	—	—	—	—	Srivastava et al. (2022)
Wang X	2024	CSF	RT‐QuIC	92%	100%	7.8%	—	11	0	1	55	Wang et al. (2024)
Wang X	2024	CSF	RT‐QuIC	95%	100%	4.9%	—	19	0	1	15	Wang et al. (2024)
Bowen S	2025	CSF	α‐Synuclein SAA	55.20%	96.30%	9.67%	4.82%	16	1	13	26	Bowen et al. (2025)
Oftedal L	2023	CSF	α‐Synuclein SAA	82.6%	88.2%	—	—	100	6	21	45	Oftedal et al. (2023)
Siderowf A	2023	CSF	α‐syn SAA	86%	96.3%	—	—	44	6	7	157	Siderowf et al. (2023)
Middleton J S	2023	CSF	α‐Synuclein SAA^a^	92.70%	96.30%	3.50%	1.80%	51	4	4	105	Middleton et al. (2023)
Concha‐Marambio	2023	CSF	α‐Synuclein SAA	98%	—	—	—	—	—	—	—	Concha‐Marambio et al. (2023)
Concha‐Marambio	2023	CSF	α‐Synuclein SAA	93%	—	—	—	—	—	—	—	Concha‐Marambio et al. (2023)
Poggiolini I	2022	CSF	RT‐QuIC	89%	96%	—	—	66	2	8	53	Poggiolini et al. (2022)
Bargar C	2021	CSF	RT‐QuIC	98%	100%	—	—	143	0	3	68	Bargar et al. (2021)
Pinder P M	2021	CSF	RT‐QuIC	100%	90.50%	0%	1.90%	20	22	0	209	Pinder et al. (2021)
Russo M J	2021	CSF	α‐Synuclein SAA	89.30%	100%	5.80%	0%	25	0	3	30	Russo et al. (2021)
Orrù C D	2021	CSF	RT‐QuIC	95%	98%							Orrù et al. (2021)
Kurihara M	2024	CSF	RT‐QuIC	58%	91%	—	—	29	5	21	50	Kurihara et al. (2024)
Kurihara M	2024	CSF	RT‐QuIC	52%	95%	—	—	22	1	20	18	Kurihara et al. (2024)
Kurihara M	2024	CSF	RT‐QuIC	88%	90%	—	—	7	2	1	18	Kurihara et al. (2024)
Rossi M.	2020	CSF	RT‐QuIC	95.20%	98.00%	—	—	20	2	1	99	Rossi et al. (2020)
Singer W	2020	CSF	NFL ELISA	97%	90%	—	—	—	—	—	—	Singer et al. (2020)
Singer W	2020	CSF	PMCA	97%	100%	—	—	—	—	—	—	Singer et al. (2020)
Singer W	2020	CSF	PMCA	100%	93%	—	—	—	—	—	—	Singer et al. (2020)
Manne S	2019	CSF	RT‐QuIC	100%	100%	—	—	—	—	—	—	Manne, Kondru, Hepker, et al. (2019)
Wang X.	2024	CSF	RT‐QuIC	75%	94%	—	—	64	5	21	74	Wang et al. (2024)
Ning H	2019	CSF	PMCA	85.30%	91.40%	—	—	—	—	—	—	Ning et al. (2019)
Shahnawaz M	2017	CSF	PMCA	88.20%	93.80%	3.90%	3.00%	67	4	9	61	Shahnawaz et al. (2017)
Schaeffer E	2024	Blood	α‐Synuclein SAA	98.80%	100%	1.20%	0%	79	0	1	20	Schaeffer et al. (2024)
Wang, Z	2024	Blood	α‐Synuclein SAA	80.50%	90.50%	4.60%	4.50%	66	4	16	38	Wang et al. (2024)
Yan S	2024	Blood	L1EV	74%	67%	—	—	—	—	—	—	Yan et al. (2024)
Emmi A	2025	Skin	RT‐QuIC	66.70%	96.20%	10.82%	3.25%	14	2	7	50	Emmi et al. (2025)
Kuang Y	2024	Skin	QSAA	90.20%	91.40%	—	—	193	18	21	190	Kuang et al. (2024a)
Wang Z	2024	Skin	RT‐QuIC	80.49%	95.35%	—	—	67	2	16	41	Wang et al. (2024a)
Wang Z	2024	Skin	RT‐QuIC	75.00%	100%	—	—	66	0	22	43	Wang Z et al. (2024a)
Wang Z	2024	Skin	RT‐QuIC	100%	100%	—	—	24	0	0	10	Wang et al. (2024b)
Dellarole I L	2024	Skin	Tau SAA	73%	93%	—	—	—	—	—	—	Dellarole et al. (2024)
Martinez‐Valbuena	2022	Skin	QSAA	90.20%	91.40%	2.10%	1.90%	193	18	21	190	Martinez‐Valbuena et al. (2022)
Zheng Y	2023	Skin	α‐Synuclein SAA	91%	92%	2.00%	2.00%	—	—	—	—	Zheng Y et al. (2023)
Martinez‐Valbuena	2022	Skin	RT‐QuIC	87%	85%	—	—	20	4	3	23	Martinez‐Valbuena et al. (2022)
Martinez‐Valbuena	2022	Skin+Blood	RT‐QuIC	84.60%	85%	9.90%	8.00%	11	3	2	17	Martinez‐Valbuena et al. (2022)
Mammana A	2021	Skin	α‐Synuclein SAA	89%	95%	—	—	—	—	—	—	Mammana et al. (2021)
Wang Z	2021	Skin	α‐Synuclein SAA	100%	100%	—	—	20	0	0	10	Wang Z et al. (2021)
Wang Z	2021	Skin	RT‐QuIC	93%	93%	—	—	53	5	4	68	Wang Z et al. (2021)
Wang Z	2021	Skin	PMCA	80%	90%	—	—	16	2	4	19	Wang Z et al. (2021)
Wang Z	2021	Skin	RT‐QuIC	94%	98%	—	—	44	1	3	42	Wang Z et al. (2021)
Bargar C	2021	Skin+Saliva	RT‐QuIC	100%	100%	—	—	3	0	0	3	Bargar et al. (2021)
Manne S	2020	Skin	α‐Synuclein SAA	96%	96%	—	—	24	1	1	24	Manne et al. (2020a)
Manne S	2020	Skin	α‐Synuclein SAA	75%	83%	—	—	9	2	3	10	Manne et al. (2020b)
Manne S	2020	Skin	RT‐QuIC	96%	96%	3.90%	3.90%	24	1	1	24	Manne et al. (2020b)
Manne S	2020	Skin	RT‐QuIC	75%	83%	—	—	—	—	—	—	Manne et al. (2020b)
Manne S	2020	Skin	RT‐QuIC	100%	94%	—	—	16	1	0	15	Manne et al. (2020b)
Manne S	2019	Skin	RT‐QuIC	100%	93.80%	0%	6.10%	13	1	0	15	Manne, Kondru, Hepker, et al. (2019)
Emmi A	2025	GI Tract	RT‐QuIC	40.90%	95.20%	10.97%	6.05%	9	1	13	20	Emmi et al. (2025)
Emmi A	2025	GI Tract	RT‐QuIC	68.40%	90.50%	11.22%	7.45%	13	2	6	19	Emmi et al. (2025)
Vascellari S	2023	GI Tract	RT‐QuIC	95.70%	100%	4.20%	0%	22	0	1	6	Vascellari et al. (2023)
Shin C	2022	GI Tract	RT‐QuIC	10%	100%	6.70%	0%	2	0	18	20	Shin et al. (2022)
Bregendahl M	2024	Saliva	α‐Synuclein SAA	67.30%	90.30%	—	—	—	—	—	—	Bregendahl et al. (2024)
Zheng Y	2023	Saliva	α‐Synuclein SAA	51%	91%	5.90%	3.10%	—	—	—	—	Zheng et al. (2023)
Zheng Y	2023	Saliva	α‐Synuclein SAA	79%	88%	4.10%	4.60%	—	—	—	—	Zheng et al. (2023)
Stefani A	2021	Saliva	RT‐QuIC	45.20%	89.80%	—	—	47	6	57	53	Stefani et al. (2021)
Manne S	2020	FFPE sections	RT‐QuIC	76%	100%	—	—	12	0	4	16	Manne et al. (2020a)

*Note:* Each study's full methodological details can be accessed via the referenced literature in the table.

Abbreviations and Explanations α‐Synuclein SAA: α‐Synuclein Seed Amplification Assay—A SAA specifically targeting the misfolded form of the alpha‐synuclein protein. RT‐QuIC: Real‐Time Quaking‐Induced Conversion—A specific type of SAA that uses real‐time fluorescence detection and periodic shaking to amplify seeds. PMCA: Protein Misfolding Cyclic Amplification—A specific type of SAA that uses cycles of sonication to amplify seeds. QSAA: Quantitative Seed Amplification Assay—A SAA method that provides quantitative measurement of the seed amount. iRS: intelligent Ranking System—Likely refers to a specific, proprietary algorithm or platform for analyzing SAA data. CSF: Cerebrospinal Fluid—The fluid surrounding the brain and spinal cord. GI Tract: Gastrointestinal Tract—The stomach and intestines. FFPE: Formalin‐Fixed Paraffin‐Embedded—A method for preserving tissue samples for pathological examination. BH: Brain Homogenate—Homogenized brain tissue. EVs: Extracellular Vesicles—Small particles released by cells. “Neuronal EVs” are those derived from neurons. L1CAM: L1 Cell Adhesion Molecule—A protein used as a marker to isolate neuron‐derived extracellular vesicles from blood. TP: True Positive—The number of correctly identified positive cases. FP: False Positive—The number of incorrectly identified positive cases. FN: False Negative—The number of incorrectly identified negative cases. TN: True Negative—The number of correctly identified negative cases. Sens_SE: Sensitivity Standard Error—A measure of the statistical uncertainty of the sensitivity point estimate. Spec_SE: Specificity Standard Error—A measure of the statistical uncertainty of the specificity point estimate. IP‐enhanced SAA: Immunoprecipitation‐enhanced Seed Amplification Assay—A SAA that uses an antibody step to purify and concentrate the target protein from a complex sample like blood before the amplification reaction.

^a^ α‐Synuclein SAA: α‐Synuclein Seed Amplification Assay; SAA serves as an umbrella term for ultrasensitive protein misfolding amplification technologies, with the specific methods in this study being Protein Misfolding Cyclic Amplification (PMCA) and Real‐Time Quaking‐Induced Conversion (RT‐QuIC). SAA (Seed Amplification Assay) is an umbrella term encompassing RT‐QuIC and PMCA; all methods listed in this table are subtypes of SAA.

### 3.2 Limitations of α‐Syn‐SAA in Differential Diagnosis

Despite the excellent diagnostic performance of α‐synuclein seed amplification assays (α‐syn‐SAA) in confirming PD and related Lewy body pathology, there remain prominent limitations in their application for the differential diagnosis of atypical Parkinsonism, which have become core challenges restricting the standardized clinical application of this technology.

At first, α‐syn‐SAA exhibits significant differences in diagnostic sensitivity across distinct α‐syn. For cerebrospinal fluid (CSF) based α‐syn‐SAA, the diagnostic sensitivity for PD exceeds 90%, while the sensitivity for multiple system atrophy (MSA) is only approximately 60% (Rossi 2021; Ma et al. 2024; Manne et al. 2020a). This markedly reduced sensitivity for MSA indicates that a single negative result of α‐syn‐SAA cannot effectively rule out the diagnosis of MSA, and this limitation is even more prominent in early‐stage MSA cases with relatively slight α‐synuclein pathological deposition.

In addition, inter‐study heterogeneity has a non‐negligible impact on the consistency of differential diagnosis using α‐syn‐SAA. A variety of factors, including differences in sample types, technical platforms, study populations and design schemes, as well as the potential risk of bias in some included studies, may cause variations in the estimated diagnostic efficacy of α‐syn‐SAA (Zhao et al. 2025), thereby further exacerbating clinical differential diagnosis between PD and atypical parkinsonian disorders.

More importantly, the current field lacks unified standards for the interpretation of α‐syn‐SAA results. The nonuniformity of core experimental parameters, fluorescence threshold settings, and critical value standards for positive judgment among different laboratories directly leads to the disability to replicate test results across institutions (Al‐Azzani et al. 2022; Ruf et al. 2020), thus exacerbating the instability of α‐syn‐SAA‐based differential diagnosis results in real clinical practice.

### 3.3 Conformational Variants as a Molecular Basis for Differential Diagnosis

The conformational divergence of α‐syn variants serves as the fundamental molecular underpinning for the differential diagnosis of synucleinopathies. Pathological α‐syn isolated from PD and DLB exhibits distinct conformational characteristics compared with that from multiple system atrophy (MSA), and these differences are directly manifested in the amplification kinetic properties of α‐syn‐SAA, thus acting as key molecular markers for distinguishing these two types of diseases (Grillo et al. 2025; Orrú et al. 2025).

Grillo et al. further validated this variant specific distinction in their 2025 study, which confirmed that the amplification lag time of PD‐derived α‐syn is notably shorter than that of MSA‐derived α‐syn (Grillo et al. 2025). This unique kinetic difference constitutes the core molecular mechanism for the differential identification of synucleinopathies via α‐syn‐SAA and also lays a solid foundation for the development of subsequent variant specific detection protocols tailored for different synucleinopathies.

**FIGURE 2:**
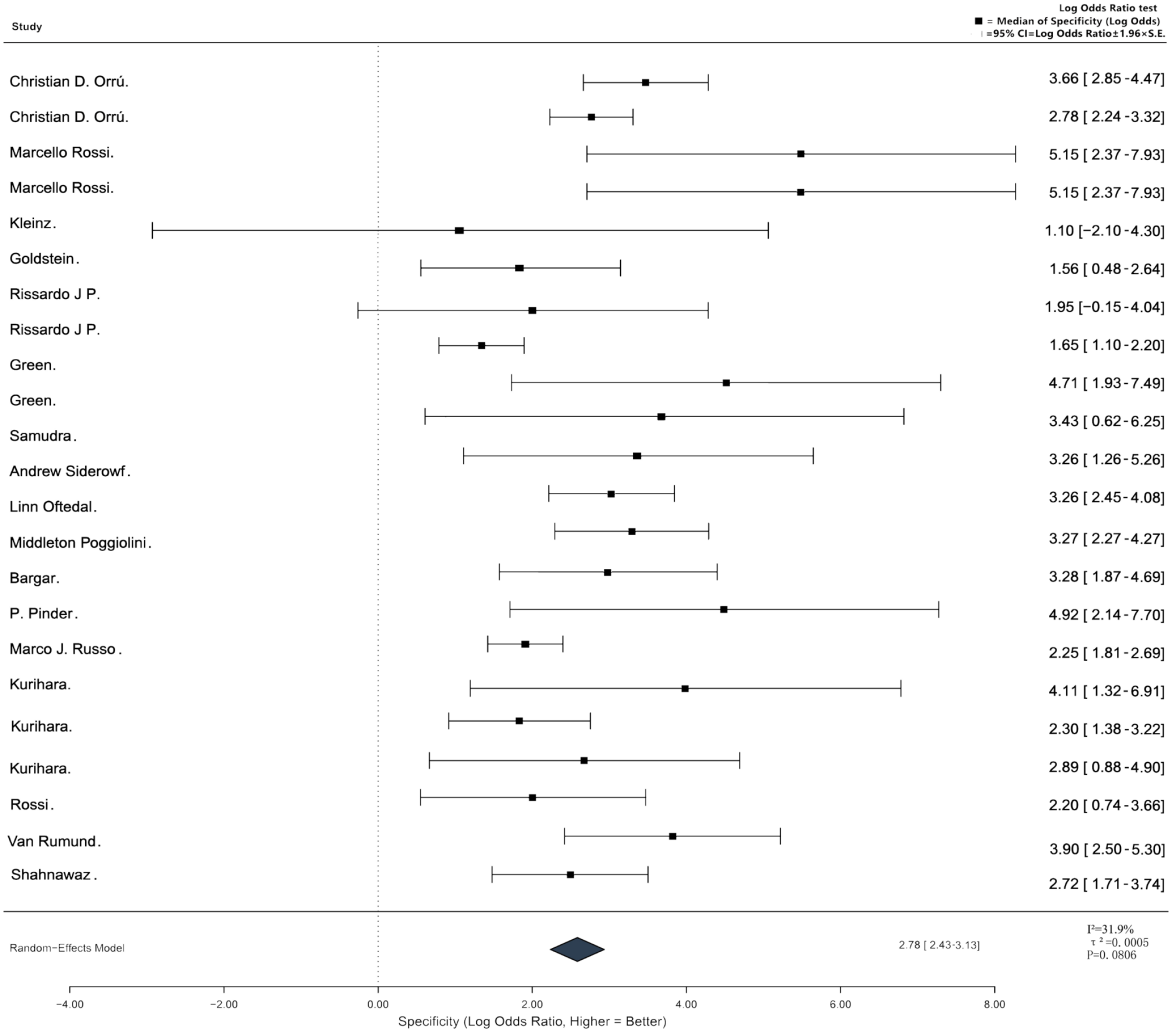
Diagnostic specificity of CSF α‐syn‐SAA: a forest plot analysis. The data were derived from 21 core studies using cerebrospinal fluid (CSF) α‐synuclein RT‐QuIC assays. Each row represents an individual study identified by the first author's name; studies from the same first author are distinguished by sequential numerical suffixes (0.1, 0.2, etc.). The square (■) indicates the median specificity expressed as a log odds ratio (Log OR), with its size proportional to the statistical weight of the study; horizontal lines represent the 95% confidence interval. The diamond (◆) represents the pooled specificity estimate derived from the random‐effects model (Log OR = 2.78, 95% CI: 2.43–3.13, which corresponds to a pooled specificity of 0.94 [95% CI: 0.92–0.96]). Heterogeneity statistics: *I* ^2^ = 31.9%, *τ* ^2^ = 0.0005, *p* = 0.0806, indicating low‐to‐moderate heterogeneity across included studies. α‐syn‐SAA, α‐synuclein seed amplification assay; CI, confidence interval; CSF, cerebrospinal fluid; Log OR, log odds ratio.

Consistent with this molecular kinetic rationale, Figure 2 summarizes the diagnostic specificity of CSF α‐syn‐SAA across independent studies. The random effects model yields a pooled log odds ratio for specificity of 2.78 (95% CI: 2.43–3.13), indicating that CSF α‐syn‐SAA achieves excellent and stable diagnostic performance in differentiating α‐synucleinopathies (PD/DLB) from non‐α‐synucleinopathy parkinsonian disorders (e.g., MSA).

This high specificity directly reflects the variant specific amplification properties of α‐syn‐SAA: the conformational divergence between PD/DLB and MSA variants translates into distinct kinetic signatures that enable precise molecular discrimination, thereby validating the superiority of CSF α‐syn‐SAA as a targeted differential diagnostic tool over conventional phenotype‐based approaches.

Beyond the molecular kinetic features revealed by α‐syn‐SAA, histological characteristics also provide valuable auxiliary evidence for differential diagnosis. Specifically, the distinct distribution patterns of phosphorylated α‐syn observed in skin biopsy samples can further supplement the differential diagnosis of PD and MSA (Donadio et al. 2021), and this finding also opens up new ideas and research directions for the development of noninvasive molecular diagnostic methods for the differential identification of atypical Parkinsonism.

### 3.4 Sensitivity and Specificity Across Disease Stages

The diagnostic efficacy of α‐syn‐SAA is not constant but shows significant specific differences with the disease stages of α‐syn (Wang et al. 2021). In this study, a random effects model was constructed using RStudio software to conduct a meta‐analysis of the diagnostic efficacy of α‐syn‐SAA in different biological samples. Figure 2 was plotted to visually present the effect size and heterogeneity of each study, and a Cut‐off Analysis Figure 1 was constructed to visualize the trade‐off between sensitivity and 1 − specificity across included studies (R Core Team 2025).

The combined sensitivity, specificity, and 95% confidence interval were calculated, and the sensitivity changes of α‐syn‐SAA at different pathological stages of PD were analyzed in combination with the Braak stage system. This series of analyses not only clarified the differences in diagnostic performance of α‐syn‐SAA in different biological samples but also revealed the intrinsic association between the positive rate of α‐syn‐SAA and the pathological progression of PD, providing a scientific basis for its staged clinical application.

**FIGURE 3:**
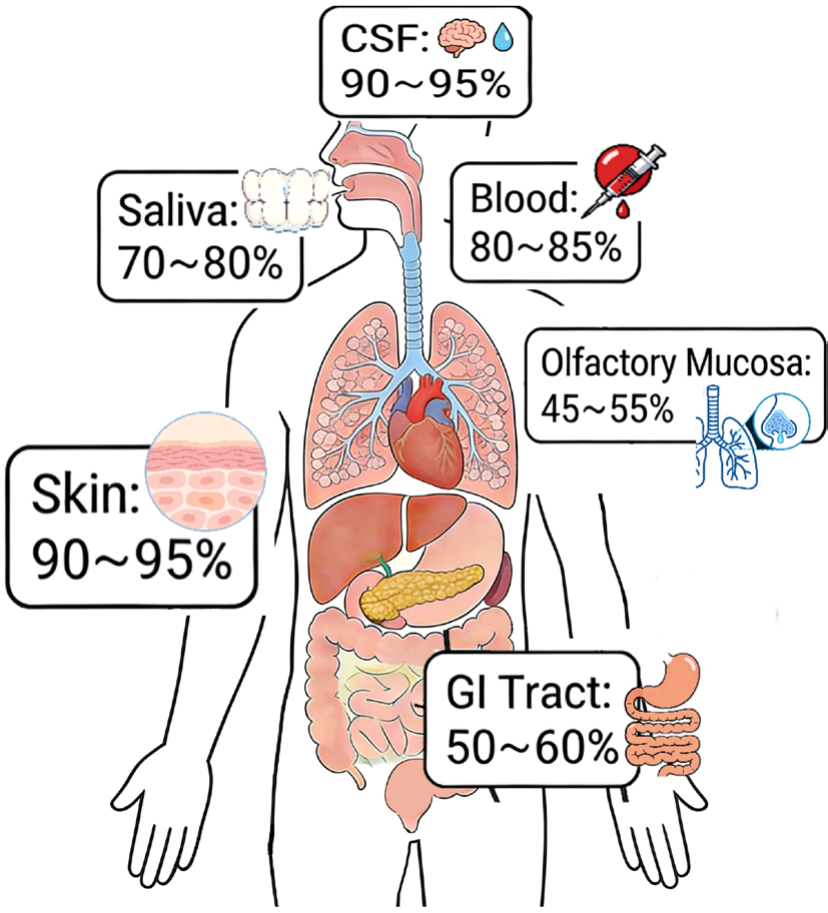
Comparative diagnostic sensitivity of α‐synuclein SAAs mapped by biological sample type. This visualization illustrates the comparative diagnostic sensitivity of α‐synuclein SAAs across six clinically relevant biological sample types, summarizing pooled data from the 112 included studies. The distribution reveals a clear diagnostic hierarchy: cerebrospinal fluid (CSF) and skin biopsies demonstrate the highest and most robust sensitivity (90%–95%), establishing them as optimal choices for definitive clinical diagnosis. Blood samples exhibit moderate‐to‐high sensitivity (80%–85%), underscoring their strategic value for large‐scale, noninvasive population screening. Conversely, while highly accessible, saliva (70%–80%), the gastrointestinal tract (50%–60%), and olfactory mucosa (45%–55%) samples show relatively lower and more variable sensitivity, positioning them currently as supplementary screening modalities. α‐syn‐SAA, α‐synuclein seed amplification assay; CSF, cerebrospinal fluid; GI, gastrointestinal.

Based on the results of a meta‐analysis of 112 included studies, α‐syn‐SAA from different biological sample sources showed significant differences in sensitivity, specificity, and clinical applicability. The sensitivity ranges of α‐syn‐SAAs across different biological samples are intuitively presented in Figure 3. It clearly shows that CSF and skin samples have the highest sensitivity (90%–95%), while olfactory mucosa samples have the lowest (45%–56%), which is consistent with the stepwise diagnostic system proposed in this study. A comprehensive comparison of the diagnostic performance, clinical advantages, and methodological challenges across these diverse biological sample types is summarized in Table 2, establishing the baseline framework for sample selection. CSF had the best diagnostic performance, while peripheral tissue samples each had their own clinical application scenarios and limitations. A specific meta‐analysis of 21 core CSF studies on CSF α‐syn RT‐QuIC showed a pooled specificity of 0.94 (95% CI: 0.92–0.96) (Grossauer et al. 2023), and there was only low‐to‐moderate heterogeneity among the studies (*I* ^2^ = 31.9%, *p* = 0.0806) (Grossauer et al. 2023), with good stability of the results.

**TABLE 2:** Comprehensive summary of diagnostic performance and application of α‐Syn SAA.

Sample types	Method	Sensitivity (range/mean)	Specificity (range/mean)	Study maturity	Advantages	Further challenges
Cerebrospinal fluid (CSF)	RT‐QuIC	93.3%–94.6%	96.3%–98.0%	High	The diagnostic performance is the best and the evidence is sufficient to can provide kinetic parameters that help distinguish disease subtypes	Collecting ways with invasive, relatively complex operation, laboratory standardization is in progress
Skin tissue	RT‐QuIC, QSAA	90.2%–91.2%	90.2%–92.0%	Medium–high	Minimally invasive, high specificity, support areas more pathological hypothesis, sampling	Sensitivity of biopsy site (highest) C7 paravertebral, tissue processing method (frozen > FFPE)
Blood/serum	RT‐QuIC	80.5% (> 95%)	~90.5%	Medium	Easy to repeat sampling, suitable for mass screening and dynamic monitoring	Significant matrix effects, requires complex pretreatment (such as exosome enrichment), and has limited ability to distinguish atypical Parkinson's disease
Saliva	RT‐QuIC	Serum: 67.3% Saliva: 74.7% A double‐positive results: 95.83%	Serum: 90.3% Saliva: 97.9% A double‐positive results: 96.15%	Exploring	Completely noninvasive, low cost and easiest to collect	Poor stability, the sampling method (vs. not stimulate), oral health, circadian rhythm, and other factors
Olfactory mucosa	RT‐QuIC	45.2%–56%	89.8%	Low to medium	It is connected to the brain and has definite pathological significance	The sampling technique is difficult, the patient's discomfort is strong, and there are age‐related physiological deposits
Gastrointestinal mucosa	PMCA	56%–95.7%	91%–100%	Low to medium	Provide direct pathological evidence for “Braak hypothesis,” has the mechanism research value	The sampling is invasive, with low patient acceptance and highly exploratory technique

CSF α‐syn‐SAA using RT‐QuIC assay has a pooled diagnostic sensitivity of 93.3%–94.6% for sporadic PD/DLB, with a specificity of 97.5%–100% (AUC = 0.96) (Mastrangelo, Caldera, et al. 2025; Bargar et al. 2021; Pilotto et al. 2023), and is currently recognized as the “gold standard” for the diagnosis of α‐syn.

Figure 1 further visualizes this high diagnostic performance, plotting sensitivity against 1 − specificity (false‐positive rate) and stratifying by detection technology (circles: RT‐QuIC; triangles: other SAA subtypes) and sample size (marker size).

RT‐QuIC assays are predominantly concentrated in the high‐sensitivity (0.80–1.00) and low‐false‐positive (0.00–0.05) region. Crucially, this visualization delineates both diagnostic accuracy and precision. While clustering closest to the ideal bottom‐right diagnostic corner reflects high overall accuracy, the tight concentration of large‐sample studies (marker size ≥ 400) underscores exceptional statistical precision. In contrast, other SAA subtypes exhibit greater dispersion, further supporting RT‐QuIC as the preferred technology for CSF‐based testing.

**FIGURE 4:**
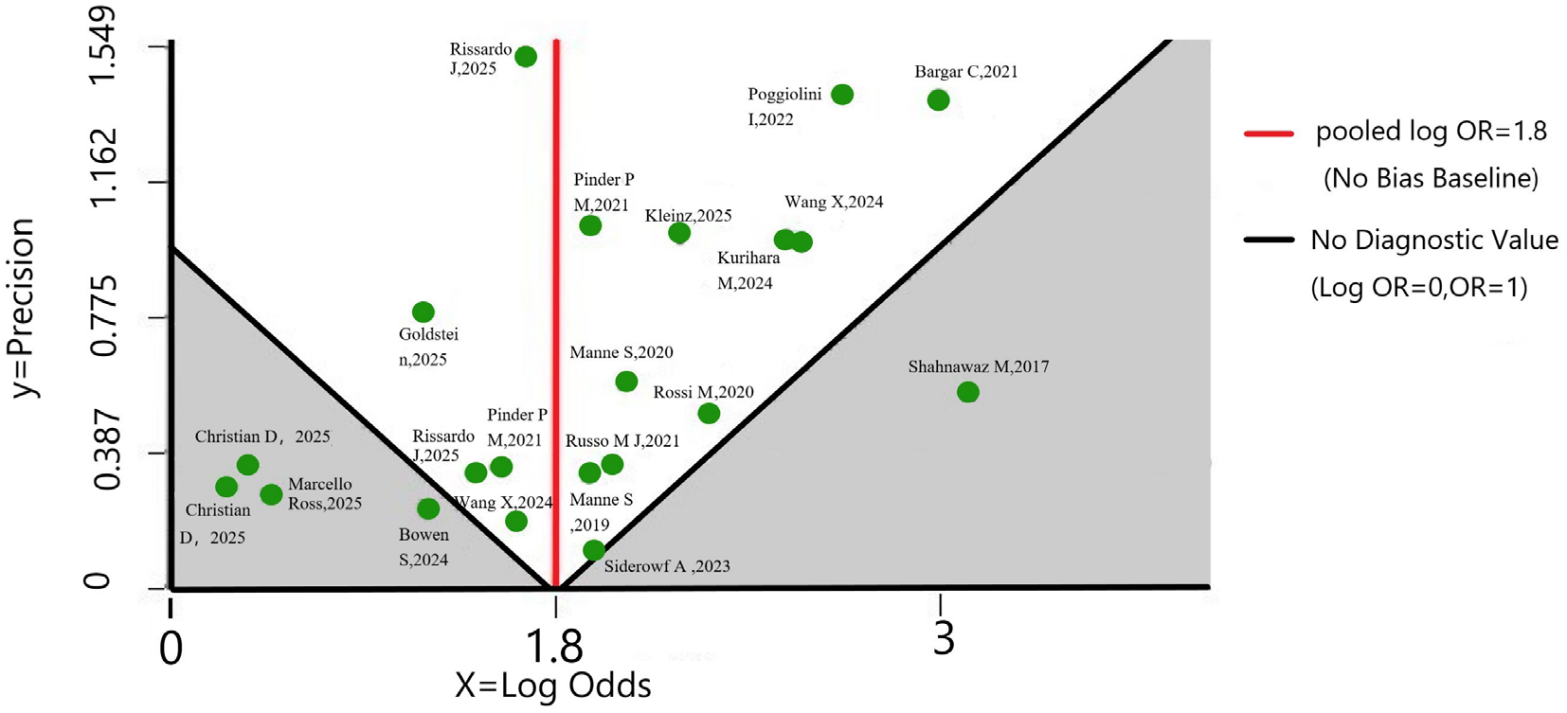
Publication bias evaluation for CSF α‐syn‐SAA sensitivity: a funnel plot analysis. Each data point represents an individual CSF‐based study, charted by its effect size (sensitivity expressed as log odds, *X*‐axis) against its statistical precision (1/standard error, *Y*‐axis). The solid red vertical line delineates the pooled effect estimate (Log OR = 1.8), while the black vertical line marks the baseline of no diagnostic value (Log OR = 0). The overall distribution of studies forms an approximately symmetrical inverted funnel around the pooled estimate, strongly indicating the absence of substantial publication bias. While minor asymmetry at the upper portion warrants cautious interpretation, this largely symmetrical clustering fundamentally validates the robustness and reliability of the reported diagnostic sensitivity.

This visual representation of precision logically extends to Figure 4, where the *Y*‐axis quantitatively represents statistical precision (the inverse of the standard error, 1/SE). Because precision is mathematically driven by sample size, the large‐scale RT‐QuIC studies clustered in Figure 1 seamlessly correspond to the high‐precision data points located at the apex of the funnel plot. Based on the log odds ratio of sensitivity, the funnel plot displays an approximately symmetrical inverted funnel distribution of all included CSF studies around the pooled Log OR = 1.8 (red solid line), with no significant outliers crossing the no‐diagnostic‐value baseline (Log OR = 0). This symmetrical distribution confirms the absence of substantial publication bias, validating that the reported high accuracy and precision of CSF α‐syn‐SAA are statistically robust and not overestimated, which strongly supports its clinical translation.

### 3.5 Improved Diagnostic Accuracy via Combined Detection

Given the limitations of a single α‐syn‐SAA in differential diagnosis, combined detection has emerged as a core strategy to enhance the diagnostic accuracy of α‐synucleinopathies (Quadalti et al. 2021). This multimodal approach integrates α‐syn‐SAA with complementary biomarkers, clinical imaging, or other diagnostic techniques, effectively compensating for the shortcomings of single‐modal detection and significantly improving the precision of distinguishing PD from atypical parkinsonian disorders.

For the differentiation between PD and PSP/CBS, a single CSF‐based α‐syn‐SAA achieves a specificity of approximately 95% (Quadalti et al. 2021). However, when combined with neurofilament light chain (NfL) detection—a biomarker elevated in atypical parkinsonian disorders—the specificity can be further increased to 100%, with the AUC reaching 0.97 (Quadalti et al. 2021). This synergistic effect arises from the complementary pathological insights: α‐syn‐SAA targets pathological α‐synuclein aggregation (the core feature of PD), while NfL reflects neuronal damage that is more severe in PSP/CBS, enabling precise molecular and pathological stratification.

While CSF‐α‐syn‐SAA maintains a high overall specificity of 97.5% against non‐synucleinopathies, its relatively low sensitivity for MSA (approximately 60%) makes binary results insufficient for precise differentiation between PD and MSA. This necessitates combination with other diagnostic tools for comprehensive judgment (Rossi 2021; Manne et al. 2020a). The specific advantages, inherent limitations, and optimal application scenarios of different detection technologies for differentiating PD from MSA are comprehensively outlined in Table 3. Clinical imaging modalities such as cranial MRI play a crucial role here: MSA is often accompanied by characteristic structural changes (e.g., MSA‐C and MSA‐P), which can be integrated with α‐syn‐SAA results to rule out PD (Bellomo et al. 2025). Additionally, serum NfL levels in MSA patients are twice as high as those in PD patients (Bellomo et al. 2025), and this quantitative difference serves as an important supplementary biomarker for differentiation. The combination of α‐syn‐SAA, cranial MRI, and serum NfL not only enhances the specificity of PD‐MSA differentiation but also improves the detection rate of early MSA cases with subtle pathological changes.

**TABLE 3:** The advantages and disadvantages of different detection techniques in the diagnosis of PD and MSA.

Detection technology	Samples	PD efficacy sensitivity/specificity	MSA efficacy sensitivity/specificity
RT‐QuIC	CSF	82.6%–89%/88.2%–96%	2.5%–75%/96%–99%
αSyn SAA	CSF	95%/95%	84%–91%/68%–87%
RT‐QuIC	Frozen skin (C7 paravertebral biopsy)	94%–96%/96%–100%	—
αSyn SAA	Oral mucosa	67.3%/90.3%	53.5%
Combined detection (RT‐QuIC + NfL)	CSF/plasma	91.4%/99%	99%

Detection technology	Advantages	Disadvantages	Application scenarios
RT‐QuIC	Sufficient clinical evidence; rapid (30–100 h)	MSA sensitivity varies greatly; invasive sampling	Early PD diagnosis; CSF initial screening
αSyn SAA	Distinguishes pathological hallmarks; high MSA sensitivity	MSA specificity low (68%); limited clinical adoption	MSA differential diagnosis; research subtyping
RT‐QuIC	Minimally invasive; stable frozen sample results	FFPE sample sensitivity reduced (75%); biopsy site‐dependent	CSF‐unavailable alternative; PD long‐term monitoring
αSyn SAA	Noninvasive; simple sampling	Moderate efficacy for both; no MSA specificity data	Noninvasive screening
Combined detection (RT‐QuIC + NfL)	High diagnostic value; low misdiagnosis rate	Higher cost; invasive CSF collection	Ambiguous parkinsonism confirmation

Notably, the value of combined detection also lies in addressing the limitations of intermediate results in α‐syn‐SAA testing. For patients with ambiguous α‐syn‐SAA outcomes (accounting for 5%–12% of tests; Iranzo et al. 2021), supplementary detection of NfL, DAT‐PET, or MIBG scintigraphy can help clarify the diagnosis, reducing the risk of overdiagnosis or missed diagnosis (Magalhães and Lashuel 2022). Quadalti et al.'s (2021) study further confirmed that the multimodal diagnostic system, centered on α‐syn‐SAA and supplemented by NfL and clinical imaging, significantly reduces the misdiagnosis rate of atypical parkinsonian disorders in primary medical institutions (from 25%–30% to < 5%) (Quadalti et al. 2021; 2023), highlighting its practical clinical value.

### 3.6 Accurate Identification of α‐Syn‐Related Proteinopathy in Atypical Parkinsonism via Seed Amplification Assay (SAA)

A major diagnostic challenge in the field of α‐synucleinopathies lies in the high misdiagnosis rate between typical forms, such as Parkinson's disease (PD), and atypical parkinsonism, including multiple system atrophy (MSA). This issue is primarily driven by overlapping clinical phenotypes and the inherent limitations of traditional, phenotype‐based diagnostic approaches.

Clinically, PD and MSA exhibit an overlap rate of up to 40% in early clinical symptoms, with nonspecific manifestations including unsteady gait and autonomic dysfunction being common to both diseases (Di Spiezio et al. 2021). This significant phenotypic overlap directly leads to a misdiagnosis rate of 25%–30% for PD and MSA in primary medical institutions, creating great obstacles for the early and accurate diagnosis of such diseases in primary clinical practice. The diagnostic predicament is further exacerbated in atypical clinical cases: for PD patients presenting with early cognitive impairment and MSA patients dominated by parkinsonian symptoms, it is difficult to achieve precise differential diagnosis merely by relying on clinical phenotypic characteristics and routine auxiliary examinations (Bougea et al. 2025).

This clinical reality fully highlights the urgent clinical need for specific molecular diagnostic methods and also underscores the necessity and clinical value of applying α‐syn‐SAA in the differential diagnosis of α‐synucleinopathies and other atypical parkinsonian disorders.

## 4 Discussion

### 4.1 Stage‐Specific Sensitivity of α‐Syn‐SAA Based on Braak Stage

Braak stage serves as the core system for describing PD pathological progression, clearly illustrating the gradual spread of α‐syn from peripheral tissues to the CNS (Braak and Del Tredici 2004; Visanji et al. 2013). Notably, the sensitivity of α‐syn‐SAA exhibits distinct stage‐specificity corresponding to Braak stage, with peripheral and central samples demonstrating complementary diagnostic value across different pathological stages.

In Braak Stages 1–2 (prodromal PD), α‐syn is primarily deposited in peripheral tissues, olfactory bulb, and lower brainstem, with no typical clinical motor symptoms yet emerged. At this stage, peripheral samples offer superior diagnostic sensitivity: the sensitivity of skin biopsy SAAs reaches 66.6% (Wang et al. 2021), and the pooled sensitivity of olfactory mucosa ranges from 45% to 56% (De Luca et al. 2019). Combined detection of two or more peripheral samples can further elevate the sensitivity to 83% (Wang et al. 2021), whereas the sensitivity of CSF samples is merely 48% (De Luca et al. 2019), making it challenging to achieve effective detection of prodromal PD.

As the disease progresses to Braak Stages 3–4 (early clinical PD), α‐syn invades the substantia nigra and midbrain, accompanied by the onset of typical motor symptoms. At this juncture, the diagnostic sensitivity of CSF RT‐QuIC exceeds 90% (Srivastava et al. 2022), becoming a core tool for early clinical PD diagnosis with the ability to stably identify the disease state.

In Braak Stages 5–6 (advanced PD), α‐syn is widely deposited in the cerebral cortex and limbic system, with extensive pathological dissemination. For advanced PD, the detection sensitivity of SAAs in both CSF and skin samples remains above 95% (Wang et al. 2021), which is highly consistent with the extensive pathological spread characteristic. This stable diagnostic efficacy enables α‐syn‐SAA to be used for dynamic assessment of disease progression in advanced stages.

### 4.2 Diagnostic Performance and Limitations of α‐Syn‐SAA in the Prodromal Stage

The prodromal stage represents a critical window for PD disease‐modifying treatment, as early intervention during this period can maximize the protection of dopaminergic neurons and delay disease progression (Carrazana et al. 2025). However, current diagnostic methods suffer from obvious deficiencies in sensitivity and specificity for prodromal PD, making early identification and intervention challenging.

Idiopathic rapid eye movement sleep behavior disorder (iRBD) is widely recognized as the core prodromal population for PD, and α‐syn‐SAA has emerged as the most promising molecular biomarker for prodromal PD detection, exhibiting substantial disease predictive value in the iRBD cohort—especially when combined with other prodromal biomarkers to enhance risk stratification accuracy. Table 4 systematically compares the diagnostic performance of SAA subtypes across different biological samples between the clinical and prodromal stages, highlighting the stage‐specific advantages of each screening modality.

**TABLE 4:** Comparison of diagnostic performance of different αSyn‐SAA assays for PD clinical versus prodromal stages.

Biological samples	Detection technology	Sensitivity (95% CI) (clinical stage, PD patients)	Specificity (95% CI) (clinical stage, PD patients)	Sensitivity (95% CI) (prodromal stage, iRBD/hyposmia/high‐risk population)	Specificity (95% CI) (prodromal stage, iRBD/hyposmia/high‐risk population)
Cerebrospinal fluid	αSyn SAA (RT‐QuIC)	(1) 82.6% (2) 98%	(1) 88.2% (76.1%–95.6%) (2) 100% (93.4%–100%)	(1) 86% (84.9%–90.5%) (2) 88% (3) 93%	(1) 96.3% (93.4%–99.2%) (2) 83%
Skin (posterior cervical C7 paravertebral/leg biopsy)	αSyn SAA (RT‐QuIC)	(1) 95% (77%–100%) (2) 96%	(1) 100% (84%–100%) (2) 96%	66.6%	96.2%
Skin (frozen)	αSyn SAA (RT‐QuIC)	96%	96%	N.A.	N.A.
Olfactory mucosa	αSyn SAA (RT‐QuIC)	(1) 46.3% (2) 67.3%	(1) 89.8% (2) 90.3%	(1) 44.4% (2) 63.6%	89.8%
Serum + Saliva	αSyn SAA (RT‐QuIC)	95.83%	96.15%	N.A.	N.A.

Abbreviation: N.A., not available.

In contrast to the marked deficiency in specificity of conventional diagnostic methods and the lack of unified quantitative validation data, Figure 2 pretreatment provides high‐reliability quantitative evidence for CSF α‐syn‐SAA in prodromal detection. It incorporates 21 global multicenter studies, with a pooled specificity of 0.94 (95% CI: 0.92–0.96) via the random effects model, accompanied by low‐to‐moderate heterogeneity (*I* ^2^ = 31.9%, *p* = 0.0806) and a near‐zero *τ* ^2^ value, confirming the universal clinical applicability of its high specificity.

For hospitals and laboratories, this translates to a misdiagnosis rate controlled within 6%—far superior to conventional methods—and the plot clarifies study weight and result stability, enabling direct reference to standardized detection efficacy data to improve diagnostic normativity.

A 2021 longitudinal cohort study by Iranzo et al. showed that the baseline positive rate of CSF α‐syn RT‐QuIC in the iRBD population ranged from 86% to 93.1% (Iranzo et al. 2021), and longitudinal follow‐up data indicated that 40%–60% of iRBD patients positive for CSF‐SAA would convert to typical synucleinopathies (PD/DLB) within 6 years (Mastrangelo, Mammana, et al. 2025; Rossi et al. 2021). For minimally invasive skin samples, the study demonstrated that skin SAA had a positive predictive value of 76% and a negative predictive value of 67% for revealing underlying pathological α‐synuclein in iRBD patients, supporting its utility as a diagnostic tool for prodromal synucleinopathies (Liguori et al. 2023).

Beyond single‐marker detection, combined application of α‐syn‐SAA with other prodromal biomarkers significantly improves PD risk stratification precision: iRBD patients positive for SAA plus reduced cardiac MIBG uptake have a 3.8‐fold higher risk of disease conversion than those with a single positive indicator (Rissardo et al. 2025), while those with SAA positivity combined with elevated NfL face a 2–3 times higher risk of PD conversion compared to patients with isolated SAA positivity (Brown et al. 2025). Additionally, the integration of α‐syn‐SAA with prodromal manifestations such as anosmia further supports the early identification of high‐risk populations.

Conventional diagnostic methods fail to balance sensitivity and specificity effectively and lack intuitive visual validation for technical efficacy differences, Figure 1 clearly demonstrates the superiority of α‐syn‐SAA—especially RT‐QuIC—via an ROC scatter plot. RT‐QuIC's circular scatter points cluster in the optimal diagnostic area (sensitivity: 0.80–1.00, 1 − specificity: 0.00–0.05), with larger‐sample studies showing more robust performance, while other SAA subtypes exhibit result dispersion.

For laboratories, the plot identifies RT‐QuIC as the preferred technique, provides direct reference for setting positive judgment cut‐off values, and its large‐sample stable results offer a replicable standard for standardized detection, improving the comparability of interinstitutional test results.

Despite these promising performances, the application of α‐syn‐SAA in prodromal PD diagnosis still faces multiple core limitations that restrict its practical utility.

Most relevant studies adopt single‐center, small‐sample designs, which may overestimate diagnostic efficacy; notably, the quantitative relationship between α‐syn seed load changes and disease progression rate remains unclear, and large‐sample, multicenter longitudinal validation data are lacking, resulting in poor result extrapolation (Wang et al. 2021).

Research on conventional prodromal PD detection is often compromised by significant publication bias, impairing result credibility, but Figure 4 verifies the robustness of α‐syn‐SAA's sensitivity results methodologically. The 21 included studies show an approximately symmetrical inverted funnel distribution with no significant publication bias, and a pooled Log OR = 1.8 corresponding to a clinical sensitivity of over 85.8%.

For hospitals and multicenter laboratory collaborations, this validates the reliability of α‐syn‐SAA for cross‐institutional prodromal screening, allowing laboratories to apply this detection to large‐sample population screening with confidence and improving the extrapolation of research and clinical diagnosis results.

Moreover, intermediate results account for 5%–12% of α‐syn‐SAA tests in the population at the prodromal stage (up to 12% for blood samples and 5% for CSF samples) (Iranzo et al. 2021); however, there is currently no unified guideline for interpreting intermediate results, creating significant confusion for clinical diagnosis. Furthermore, the value of single α‐syn‐SAA testing for prodromal risk stratification is limited, and standardized multi‐marker combined detection protocols are still lacking in clinical practice, making precise stratification of the prodromal population difficult.

More importantly, even with effective identification of prodromal PD via α‐syn‐SAA, translating these detection results into effective early intervention measures is hindered by a series of practical barriers. On the one hand, the lack of standardized procedures for α‐syn‐SAA—including non‐uniform experimental parameters, fluorescence thresholds, and critical value standards—makes it difficult to replicate test results across laboratories (Al‐Azzani et al. 2022; Ruf et al. 2020), hampering the establishment of unified diagnostic criteria for the prodromal stage.

On the other hand, Bowen et al.'s (2025) study pointed out that clinicians' insufficient ability to integrate and interpret SAA results with clinical phenotypes also affects the formulation and implementation of early intervention plans. Additionally, most disease‐modifying treatments (such as α‐syn aggregation inhibitors) are still in Phase II clinical trials (Carrazana et al. 2025), and there is a lack of treatment options matching prodromal diagnostic markers, further exacerbating the disconnection between diagnosis and intervention and becoming a significant obstacle to early intervention in prodromal PD.

## 5 Anti‐Synuclein Therapy and the “Noninvasive Gap”

The emergence of α‐syn‐SAA enables direct detection of pathological α‐syn, propels the diagnosis of α‐syn from traditional total α‐syn concentration detection to specific molecular detection, and lays the foundation for the development of anti‐synuclein therapy.

With the continuous development and advancement of anti‐synuclein antibody therapy, the treatment of α‐syn is gradually moving toward early and precise, but the existence of the “noninvasive gap” makes it difficult to effectively carry out key links such as the screening of the treatment target population, efficacy monitoring, and stratification of clinical trial subjects.

This study, in combination with the clinical implementation challenges of α‐syn‐SAA, deeply analyzed the importance of noninvasive diagnostic methods for anti‐synuclein treatment, and also revealed the realistic contradiction between current diagnostic techniques and treatment needs, providing a direction for the subsequent development of noninvasive diagnostic methods that meet treatment needs.

### 5.1 Imperative of Bridging the “Noninvasive Gap” for Anti‐Synuclein Therapy

Filling the “noninvasive gap” has become an urgent need for the clinical development and application of anti‐synuclein therapy, and noninvasive, highly sensitive molecular diagnostic methods are the core prerequisite for achieving precise treatment of α‐syn. In terms of screening for the treatment population, anti‐synuclein treatments such as α‐syn aggregation inhibitors are mostly in Phase II clinical trials (Carrazana et al. 2025), and their therapeutic effects are best in the prodromal stage of the disease, which requires early identification of the prodromal/high‐risk population, while invasive CSF sampling cannot meet the demand for large‐scale screening of the treatment population.

In terms of efficacy monitoring, disease‐modifying treatments require long‐term longitudinal tracking of pathological changes in α‐syn, and repetitive CSF sampling cannot be achieved in clinical practice, and noninvasive biomarkers are urgently needed to monitor drug efficacy (such as changes in seed load) (Bellomo et al. 2025).

In terms of stratification of clinical trial subjects, α‐syn‐SAA can serve as a pharmacodynamic biomarker for clinical trials, but the lack of noninvasive, highly sensitive detection means makes precise stratification of trial subjects (such as prodromal/early clinical PD) difficult to achieve, directly affecting the interpretation of clinical trial results and the drug development process.

### 5.2 Clinical Translational Challenges of α‐Syn‐SAA Restricting Therapy

The clinical translational challenges of α‐syn‐SAA itself further the problems caused by the noninvasive gap, becoming an important constraint on the clinical translation of anti‐synuclein therapy. First, the lack of standardization is a core bottleneck.

Al‐Azzani et al. (2022) pointed out that the non‐uniformity of experimental parameters such as recombinant α‐syn preparation, fluorescence threshold, reaction buffer composition, and the absence of sample‐specific threshold standards led to non‐repeatable test results from different laboratories (Al‐Azzani et al. 2022; Ruf et al. 2020), making it difficult to establish a uniform screening standard for the treatment‐applicable population. Secondly, the interpretation of intermediate results remains unresolved.

Intermediate results account for 5%–12% of SAA tests (up to 12% for blood samples and 5% for CSF samples) (Iranzo et al. 2021), and there is currently no unified interpretation guideline, which may lead to overdiagnosis or missed diagnosis and affect the rational formulation of treatment plans. Again, the clinical evidence is limited, with insufficient sample‐specific validation and lack of cost–benefit analysis.

Most α‐syn‐SAA‐related studies adopt single‐center, small‐sample designs with poor result extrapolation, and large‐sample multicenter data for Asian, African, and other populations are lacking (Wang, Zheng, et al. 2024).

Furthermore, CSF α‐synuclein SAA positivity has been associated with disease progression and cognitive decline in longitudinal cohort studies (Tosun et al. 2024). Meanwhile, peripheral samples—the core of noninvasive screening—have inherent diagnostic limitations: skin tissue tested by RT‐QuIC/QSAA has a combined accuracy of 94.1% and a stable specificity of 90.2%–92.0% (Donadio et al. 2021; Kuang, Mao, Gan, et al. 2024), but its sensitivity is markedly affected by biopsy site and tissue treatment (as systematically detailed in Table 5), with the left thigh (69.2%) and calf (68.1%) being optimal and neck sites showing lower sensitivity (60.4%) (Emmi et al. 2025); unenriched blood/plasma has a pooled sensitivity of ~80.5% and specificity of ~90.5% (Wang, Gilliland, et al. 2024; Zubelzu et al. 2022; Li et al. 2024; Tsao et al. 2022; Wang et al. 2022), and matrix interference necessitates pretreatment such as neuron‐derived exosome enrichment, yet the 95.8% sensitivity achieved by combined serum and saliva detection in a European cohort (Wang, Zheng, et al. 2024) lacks validation in diverse populations; saliva/olfactory mucosa samples are easily accessible but highly unstable (saliva degrades within 2 h at room temperature; Vivacqua et al. 2016) and low in sensitivity (70%–80% and 45%–55% respectively; Emmi et al. 2025; Kuang, Mao, Gan, et al. 2024; Zheng et al. 2024; Chahine et al. 2023), making them only suitable for auxiliary screening in resource‐limited settings and unable for standalone α‐syn diagnosis.

**TABLE 5:** Comparison of positive detection rates of RT‐QuIC in different tissue sites.

Anatomical biopsy sites	Location	Positive/detections (%)	Replication well positive (%)		Lag time (hours), mean ± SD
Optimal anatomical biopsy sites	Posterior cervical (C7 paravertebral)	Sensitivity, 100% specificity Modified SAA: 92.46% Syn SAA: 87.7% (advanced PD)	SAA: 4 replicate wells design	≥ 2/4 wells as positive	Modified SAA: 5.5 ± 2.0 h SAA:30 h
Nonoptimal anatomical biopsy sites	Lower leg/thigh (distal skin)	Positive rate lower than C7 paravertebral	RT‐QuIC: slightly lower multi‐well consistency	RT‐QuIC: slightly lower multi‐well consistency	Criteria for C7 paravertebral site: marked prolongation of lag time
	Stomach	80.0% accuracy; 88.9% sensitivity (advanced PD)	4 replicate wells design	≥ 2/4 wells as positive	40 h
	Duodenum	67.4% accuracy; 58.8% sensitivity (advanced PD)	4 replicate wells design	≥ 2/4 wells as positive	40 h

Finally, Bowen et al. (2025) noted that clinicians' low familiarity with SAA technology and insufficient ability to integrate and interpret SAA results with clinical phenotypes (e.g., interpreting positive skin SAA results in patients with atypical motor symptoms) limit the implementation of SAA‐guided individualized treatment.

### 5.3 Value of α‐Syn‐SAA in Anti‐Synuclein Therapy for Prodromal/Early PD

Despite numerous clinical translational challenges, α‐syn‐SAA still holds irreplaceable value in anti‐synuclein therapy for prodromal/early PD and serves as an important bridge between early diagnosis and precision treatment. α‐syn‐SAA can detect pathological changes before the onset of motor symptoms, providing a critical time window for disease‐modifying therapy, which is most effective before the massive loss of dopaminergic neurons and can delay disease progression to the greatest extent (Srivastava et al. 2022). Pereira et al. (2023) reports DOPA decarboxylase as an emerging biomarker for preclinical Lewy body disease, supporting the concept of early/preclinical detection.

The α‐syn seed load in PD patients is significantly positively correlated with UPDRS III score; a series of SAA tests can effectively distinguish between those with rapid disease progression and those with slow progression, providing guidance for individualized symptomatic treatment—early addition of dopamine receptor agonists is recommended for patients with rapid progression (Bellomo et al. 2025).

At the same time, α‐syn‐SAA can serve as a biomarker to objectively evaluate the therapeutic effects of drugs such as α‐syn aggregation inhibitors, where a decrease in seed load directly indicates therapeutic effectiveness, providing important molecular evidence for clinical trials and clinical efficacy evaluation. In addition, in prodromal populations such as iRBD, α‐syn‐SAA enables the early identification of high‐risk groups, providing precise research subjects for targeted intervention studies and treatments.

Notably, to further unleash the potential of α‐syn‐SAA in anti‐synuclein therapy for prodromal/early PD, overcome the aforementioned clinical translational challenges, and meet the clinical demand for noninvasive, efficient diagnostic tools, research has gradually shifted toward noninvasive sampling of peripheral tissues such as skin, blood, and saliva in recent years.

The core objective of this shift is to achieve sensitivity and specificity in these peripheral samples comparable to CSF testing, thereby optimizing the early diagnosis and individualized treatment of α‐syn. Based on the results of this study, future efforts should focus on advancing this noninvasive research direction: optimizing technical protocols to address matrix interference and stability issues of peripheral samples, establishing standardized procedures for noninvasive α‐syn‐SAA, and validating the performance of these noninvasive tools across diverse populations and disease stages.

This will not only enhance the accessibility of α‐syn‐SAA in clinical practice but also strengthen its role as a bridge between early diagnosis and precision therapy, ultimately maximizing the clinical value of anti‐synuclein therapy for prodromal/early PD.

### 5.4 Current Diagnostic Performance of Mainstream Noninvasive Peripheral Samples and Optimization Strategies

Among the current mainstream noninvasive peripheral samples, skin biopsy and blood samples have relatively mature diagnostic performance and are the core directions for future clinical translation, while saliva, olfactory mucosa, and gastrointestinal samples can be used as auxiliary screening methods to form a noninvasive diagnostic system with complementary multiple samples.

Skin biopsy is currently the most mature minimally invasive testing method, with a specificity of 90.2%–92.0% and a comprehensive diagnostic accuracy of 94.1% (Doppler 2021; Donadio et al. 2021; Kuang, Mao, Huang, et al. 2024). Its sensitivity can be significantly improved by optimizing the biopsy site and tissue processing methods, and when combined with other peripheral samples for detection, it is the best tool for prodromal screening (De Luca et al. 2019); however, its main limitation is that the sensitivity is still lower than that of CSF, with a risk of missing some patients.

Blood samples are the most potential noninvasive samples for large‐scale application, with a baseline sensitivity of about 80.5% and a specificity of about 90.5% (Wang, Gilliland, et al. 2024; Zubelzu et al. 2022). Pretreatment methods such as neuronal exosome enrichment have raised their sensitivity to 95.8% in the European population (Wang, Zheng, et al. 2024), but more extensive validation is still needed in non‐Western populations to address the issues of matrix interference and population adaptability.

Saliva/olfactory mucosa/gastrointestinal samples are highly convenient auxiliary screening tools, and combined detection of multiple samples can improve the efficiency of prodromal detection (De Luca et al. 2019), but the core problems of poor sample stability and low sensitivity still need to be resolved to further expand their clinical application scenarios.

To address these inherent limitations of noninvasive peripheral samples and enhance the diagnostic performance of α‐syn‐SAA, the optimization of noninvasive α‐syn‐SAA detection requires a multifaceted approach, including standardization construction, next‐generation technology research and development, and in‐depth research on the biological mechanisms of α‐syn variants, while targeting the core technical bottlenecks in current detection.

First, prioritize the construction of a standardization system. Through international multicenter cooperation, develop a unified SOPs covering core contents such as standardized production of recombinant α‐syn, reaction conditions, and consensus critical values; establish a pathologically confirmed PD/DLB/MSA reference sample library for interlaboratory calibration to reduce result variation; construct a three‐level interpretation system for intermediate results (Magalhães and Lashuel 2022), and establish an international intermediate results database to accumulate long‐term prognosis data, thereby completely resolving the clinical interpretation dilemma.

Second, optimize the core technical strategies for noninvasive α‐syn‐SAA assays and develop next‐generation detection technologies based on SAA technological evolution to break through the limitations of current noninvasive detection: IP‐QuIC and ES‐QuIC tackle matrix interference in peripheral body fluids to improve the diagnostic sensitivity of blood samples (Bregendahl et al. 2025; Di Spiezio et al. 2021); Nano‐QuIC and lysosomal QuIC shorten detection time and enhance assay convenience (Christenson et al. 2023; Brockmann et al. 2021); alternative amplification mechanisms (PMCA, QSAA) enable the detection of ultralow‐concentration seeds in peripheral samples while visualizing the spatial distribution of pathological α‐syn (Saborio et al. 2001; Mao et al. 2022).

In addition, further in‐depth research on the biological mechanisms of α‐syn variants is needed to clarify the conformational differences of PD/MSA‐derived α‐syn variants and their associations with clinical phenotypes, and to explore the molecular mechanisms of intermediate results via cryo‐electron microscopy, providing a solid molecular basis for optimizing detection protocols.

### 5.5 Clinical Translation and Application Direction of Noninvasive SAA

The clinical translation of noninvasive SAA requires the construction of a scientific application system in line with clinical needs, while strengthening translational research and health economics research to enhance the feasibility and practicality of its clinical application.

First, establish a step‐by‐step diagnostic pathway that matches the sample type with the clinical target, that is, use CSF testing for symptomatic cases, skin biopsy for minimally invasive diagnosis in high‐risk populations, and blood testing for large‐scale screening in the prodromal stage of the population to achieve a balance between diagnostic accuracy, accessibility, and patient tolerance.

This hierarchical strategy is rigorously supported by epidemiological principles. Applying highly accessible blood tests as a first‐line screen maximizes population coverage. However, because positive predictive value (PPV) inherently drops in low‐prevalence general populations, subsequent confirmatory testing using modalities with an exceptionally high positive likelihood ratio (LR+)—such as CSF or skin SAA—is strictly required for screen‐positive individuals. This step‐by‐step approach effectively mitigates the decline in PPV, optimizing comprehensive diagnostic accuracy while successfully avoiding false‐positive overdiagnosis.

Second, build a multimodal diagnostic system that integrates noninvasive SAA with biomarkers such as NfL, digital motor/cognitive assessment, Dopamine transporter positron emission tomography (DAT‐PET) and cranial MRI to make up for the limitations of a single test and further improve the accuracy of diagnosis and differential diagnosis (Quadalti et al. 2021).

Strengthen translational and health economics research to validate the value of noninvasive SAA as a pharmacodynamic biomarker for anti‐synuclein therapy clinical trials, conduct real‐world cost–benefit analyses in different medical systems, and provide evidence‐based evidence for its clinical application.

Finally, establish an international database of peripheral SAA and intermediate results, accumulate long‐term prognostic data, clarify the association between noninvasive test results and disease progression, and provide sufficient evidence‐based evidence for clinical interpretation and treatment guidance.

## 6 Research Prospects

### 6.1 Long‐Term Research Priorities

To promote the full clinical application of noninvasive SAA in the diagnosis of α‐syn and to fully leverage its core role in early diagnosis and precision treatment, a series of high‐priority research efforts will still be needed in the future.

First, conduct large‐sample, prospective multicenter clinical trials with a sample size of more than 1000 cases and a follow‐up period of more than 5 years to verify the detection efficacy of noninvasive SAA in different races and disease stages, and address the limitations of current single‐center, small‐sample studies.

Second, develop minimally invasive, highly sensitive salivary/olfactory mucosa detection techniques to address poor sample stability and low sensitivity by optimizing sample preservation and pretreatment methods, and expand the screening range in resource‐limited scenarios.

Third, explore the application value of noninvasive SAA in real‐world clinical practice to clarify its practical effects in reducing misdiagnosis rate, shortening diagnosis time, and improving patient prognosis, and to provide real‐world evidence for its clinical promotion.

Fourth, combine noninvasive SAA with artificial intelligence technology to build an automated prodromal risk stratification and treatment guidance model to achieve precise matching of diagnosis and treatment and promote the development of individualized medicine for α‐syn. Fifth, further optimize blood sample pretreatment techniques, develop standardized enrichment methods for different populations, completely solve the problem of matrix interference, and achieve comparable diagnostic efficacy of blood samples to CSF samples.

Future research should also explore the complementary value of combining α‐syn detection with SAAs and develop high‐throughput platforms such as digital SAA, verify the biological significance of multisite tissue detection to help elucidate the spatiotemporal dynamics of pathological spread, and establish long‐term follow‐up cohorts of α‐syn‐positive prodromal individuals to optimize phenoconversion prediction models.

## Author Contributions

**Weijie Kong:** methodology, investigation, writing – review and editing, writing – original draft, visualization, data curation, resources. **Mika Inada Shimamura:** writing – review and editing, resources, supervision, formal analysis, validation. **Tetsuya Maeda:** writing – review and editing, supervision. **Kenta Takahashi:** writing – review and editing, supervision. **Masanori Kurihara:** supervision, writing – review and editing. **Atsushi Iwata:** writing – review and editing, supervision. **Katsuya Satoh:** conceptualization, methodology, investigation, formal analysis, writing – original draft, writing – review and editing, supervision, funding acquisition.

## Funding

This work was supported by Japan Agency for Medical Research and Development (JP25dk0207077 and JP23ek0109620) and JSPS Grants‐in‐Aid for Scientific Research (Grant Numbers JP21K07417 and JP25K02582).

## Conflicts of Interest

The authors declare no conflicts of interest.

## Data Availability

Data sharing is not applicable to this article because no new data were created or analyzed for this systematic review and meta‐analysis. All data analyzed in this study are from previously published studies, which are cited in the reference list of the manuscript.
